# Digital Health Intervention for Patient Monitoring in Immune-Mediated Inflammatory Diseases: Cocreation and Feasibility Study of the IMIDoc Platform

**DOI:** 10.2196/58095

**Published:** 2025-04-21

**Authors:** Diego Benavent, Jose M Iniesta-Chamorro, Marta Novella-Navarro, Miguel Pérez-Martínez, Nuria Martínez-Sánchez, Mónica Kaffati, Manuel Juárez-García, Marina Molinari-Pérez, Andrea González-Torbay, Mariana Gutiérrez, Natalia López-Juanes, Victoria Navarro-Compán, Irene Monjo-Henry, Germán Rodríguez-Rosales, Javier Bachiller, Enrique Calvo-Aranda, Xabier Michelena, Laura Berbel-Arcobé, Alejandro Balsa, IMIDOC Research Team, Leticia Lojo-Oliveira, Enrique J Gómez, Chamaida Plasencia-Rodríguez

**Affiliations:** 1 Hospital Universitari de Bellvitge, L'Hospitalet de Llobregat, Carrer de la Feixa Llarga, Barcelona, 08907, Spain, 34 685351866; 2 Biomedical Engineering and Telemedicine Centre, ETSI, Center for Biomedical Technology, Universidad Politécnica de Madrid, Madrid, Spain; 3 Instituto de Investigación Hospital 12 de Octubre (imas12), Hospital Universitario 12 de Octubre, Madrid, Spain; 4 Rheumatology Department, Hospital Universitario La Paz, Madrid, Spain; 5 Kairós Digital Solutions, Madrid, Spain; 6 Obstetrics and Gynecology Department, Hospital Universitario La Paz, Madrid, Spain; 7 Clinical Immunology Department, Hospital Universitario La Paz, Madrid, Spain; 8 Orthopedics & Traumatology Department, Hospital Universitario del Henares, Madrid, Spain; 9 Rheumatology Department, Hospital Universitario Ramón y Cajal, Madrid, Spain; 10 Department of Medicine, Universidad Complutense de Madrid, Madrid, Spain; 11 Hospital Universitari Vall d’Hebron, Barcelona, Spain; 12 Centro de Investigación Biomédica en Red de Bioingeniería, Biomateriales y Nanomedicina, CIBER, Madrid, Spain

**Keywords:** immune-mediated inflammatory diseases, cocreation, health care provider, digital health solution, inflammatory, inflammatory disease, monitoring, care model, patient care, development, app, user centered, patient monitoring, decision-making, user needs

## Abstract

**Background:**

Immune-mediated inflammatory diseases, such as rheumatoid arthritis and spondyloarthritis, pose challenges due to recurrent flares and gaps in patient monitoring. Traditional health care models often fail to capture disease progression effectively.

**Objective:**

This study aimed to describes the structured cocreation of the IMIDoc platform, an interdisciplinary initiative aimed at improving patient monitoring, education, and health care provider decision-making.

**Methods:**

IMIDoc was cocreated through an interdisciplinary team involving clinical experts, biomedical engineers, and technical developers, using user-centered design principles. The development process included the identification of unmet clinical needs, user-centered app design, implementation of medication management features, patient data recording capabilities, and educational content. A 3-month feasibility and functionality testing was performed to evaluate the usability and technical performance of the apption.

**Results:**

During the feasibility testing, 111 entries were logged for the patient mobile app, comprising 76 errors identified and corrected, 16 improvements addressing functionality, usability, and performance, and 10 evolutionary suggestions. The professional interface received 45 entries, identifying 40 errors and 5 evolutionary suggestions. Ten iterative updates significantly enhanced the user interface intuitiveness and medication reminder functionality, aligning the solution closely with clinical workflows and user needs.

**Conclusions:**

The IMIDoc platform, developed by a multidisciplinary cocreation methodology, shows potential to improve the management of immune-mediated inflammatory diseases ithrough enhanced communication and monitoring. A multicenter clinical study with 360 patients across 5 Spanish hospitals will further evaluate its impact.

## Introduction

Immune‐mediated inflammatory diseases such as rheumatoid arthritis (RA) or spondyloarthritis(SpA) are chronic diseases characterized by joint or spinal inflammation with sporadic flares, leading to significant functional impairment in affected individuals [1,2]. Managing these diseases often involves complex therapeutic regimens, necessitating close monitoring and effective communication between patients and health care providers to promptly identify changes in disease progression or treatment-related concerns. Traditional care models, which rely heavily on scheduled clinical consultations, may fail to provide a comprehensive and continuous view of disease activity, resulting in gaps in patient care [3].

Advancements in digital health technologies present promising opportunities to enhance the management of immune‐mediated inflammatory diseases. Telemonitoring, in particular, offers the potential to bridge the gaps between face-to-face consultations by enabling real-time disease activity tracking and facilitating timely interventions during periods of disease instability [4]. Smartphone data collection offers enhanced research and clinical care capabilities, notably with electronic patient-reported Outcomes (ePROs) [5]. Such integration aims to optimize the quality of patient care and facilitate effective communication between health care professionals and their patients.

In this context, our research group designed the mixed care model (MAM, from the Spanish Modelo Atencion Mixta), which incorporates the use of information and communication technologies, and also designed a previous pilot study called Digireuma, with the goal of developing a digital health tool for monitoring ePROs in patients with RA and SpA[6]. The Digireuma study demonstrated that while remote monitoring via digital tools could reduce the need for physical consultations, challenges such as declining patient adherence, with around half of the patients dropping out during the follow-up of 6 months, and usability issues (primarily related to the login process), remained persistent. A targeted survey administered at the study’s conclusion, gathering insights from both patients and professionals, highlighted these challenges and helped identify critical areas for improvement.

Based on previous results, the medical team decided to develop a digital health solution specifically tailored to the needs of both health care professionals and patients. Feedback indicated a demand for reliable educational resources, leading to the development of tailored modules incorporating interactive texts, infographics, and videos created in collaboration with health care professionals. To enhance adherence, the app integrates personalized medication management tools with automated reminders and scheduled messages for patients demonstrating positive clinical evolution, thereby reinforcing a sense of continuous clinical follow‐up. In addition, bidirectional communication between patients and providers facilitates real‐time feedback, ensuring that the solution effectively bridges the gaps observed in previous digital health interventions for RA and SpA.

In the current manuscript, we delineate the collaborative effort among a clinical team, a group of biomedical engineering experts, and a dedicated team of developers, in cocreating an application tailored to the unique needs of patients with immune‐mediated inflammatory diseases. Drawing upon interdisciplinary expertise, their efforts culminated in the IMIDoc app, an innovative digital health solution, which has also led to invaluable insights and lessons for future collaborative health-tech initiatives.

## Methods

### Development of IMIDoc

In the development of our digital health app, we adopted a structured cocreation process encompassing 2 distinct phases. The initial “planning phase” involved the clinical team meticulously reviewing their previous experiences and identifying prevalent unmet needs in disease management. Concurrently, the clinical team engaged in a comprehensive assessment of potential funding strategies. Then, preliminary discussions with the engineers to outline the envisioned features and functionalities were initiated. This process culminated in the articulation of a clear statement of purpose. The subsequent “development phase*”* involved the technical and development teams, who adhered to Agile methodologies, fostering a dynamic collaboration between clinicians and engineers [7]. Central to this phase was a rigorous evaluation of pivotal factors influencing technology selection, the formulation of user profiles, as well as the definition and development of the functionalities to be incorporated into the digital solution. In addition, paramount attention was devoted to assessing the correct functioning of the digital solution by end users through pilot testing.

### Planning Phase

#### Overview With the Developers and Statement of Purpose

Upon successfully securing a sustainable funding source, we initiated consultations with several development teams, leveraging our collaboration with the biomedical engineering team from the Universidad Politécnica de Madrid. Following a public tender process, the project was awarded to Kairos DS [8]. During several collaborative sessions with Kairos DS, we meticulously crafted a statement of purpose for our digital health solution, including a description of its main functionalities and features.

#### Funding

To secure funding for a new medical app, efforts were diversified across private funding contests, collaboration with pharmaceutical companies [9,10], and exploration of public grants focused on health care innovation [11], tapping into multiple sources to maximize potential financial support.

#### Analysis of Previous Experience

An analysis of the previous remote monitoring experience, the Digireuma study [6], was conducted. For this, semistructured telephone interviews were conducted by an external expert with a randomly selected subset of patients, in order to gain a comprehensive understanding of user experiences, capturing both the strengths and potential areas for improvement of the application and implementation methodology. Clinicians who participated in the previous project completed a questionnaire to evaluate the usability of the medical interface, the usefulness of the solution and its utility in medical practice. Further information on these questionnaires can be found in Text S1 inMultimedia Appendix 1.

### Development Phase

Throughout the development phase, interdisciplinary work was shared among the clinicians, the technical software and biomedical engineering team. This also involved intensive dialogue among engineers and end-users, including both patients and clinicians.

Due to the nature of the project, Scrum was adopted by the development team and the product development stakeholders [12]. This framework is based on small, iterative cycles or sprints with each iteration adding value to the product, so that corrective measures can be taken without compromising the entire project [13].

An initial kick-off meeting between clinicians and the technical team aimed at establishing shared objectives and product expectations was held. The development schedule included 15 sprints, with each sprint ending with a review.

#### Definition of User Profiles and Functionalities

We used a user-centered design approach [14], for which by definition must consider the needs and experiences of end users via a continuous series of evaluations [15,16]. Initial user profiles were established based on interviews with clinicians and patients [6], categorizing users into health care providers and patients. For health care providers, functionalities such as patient data monitoring, alert resolution, and telematic contact were developed. For patients, features included ePROs registration, medication management, reminders, and access to educational content covering disease information, management strategies, and relevant hospital information.

#### Technical Requirements

As a starting point for designing the architecture of the system, the MAM protocol and the agents that comprise the proposed clinical intervention were considered, combining face-to-face and telematic follow-up protocols [12]. The profiles, needs and functionalities previously defined were then considered. With all of this in mind, the system and components to be developed were then defined: a mobile app for patients, an application for health professionals, and services for communication, data processing and storage.

Throughout the design and development process, it was necessary to evaluate possible technologies and their deployment (Text S2 in Multimedia Appendix 1). The system architecture was designed using cloud-based technologies, specifically Amazon Web Services (AWS), to optimize resource usage and ensure data security [17-19]. To ensure cost-effectiveness, scalability, and ease of maintenance, we selected hybrid and cross-platform technologies [20], allowing IMIDoc to be deployed on both Android and iOS platforms efficiently.

In addition, to carry out the clinical evaluation study, a series of requirements had to be met and specific documentation had to be submitted to the Information Systems Security Office of the Madrid Health Service.

### Preclinical Testing Phase of IMIDoc

During the first sprints of the project execution and once the main components and specifications of the user applications were identified, those activities associated with the interface design were carried out—that is, generating wireframes, sketches, and mock-ups. This process is key in user centered-design (UCD) and allowed us to preliminarily evaluate the structure of the applications, the composition of the scenarios and the interactions before the development phase [17].

An initial phase of testing was conducted by a multidisciplinary team of clinicians, engineers and patients, generating discussions in 3 online meetings. A subsequent digital solution testing phase with active users was planned for 4 weeks. Both patients and health care professionals were actively involved. A comprehensive guide detailing functionality, data input methods, and troubleshooting steps was provided to all participants before the testing period. The guide aimed to standardize the usage of the application for all users, thereby minimizing variation and enhancing the validity of the pilot results. During the 4-week period, feedback was collected through forms and informal interviews, focusing on usability, feature relevance, and overall experience.

### Ethical Considerations

The protocol of the study was approved by the local ethics committee, CEIm Hospital La Paz (PI-4519) and has been registered as a clinical trial (ClinicalTrials.gov Identifier: NCT06273306).

## Results

### Planning Phase

#### Scope and Main Objective of IMIDoc

In a dedicated meeting with the multidisciplinary development team, the scope of the digital solution was outlined by conducting a scoping analysis to delineate its potential capabilities and limitations. The name IMIDoc was selected for the digital health solution proposed in this project. The primary purpose of IMIDoc was defined as addressing an unmet need in the care of patients with RA and SpA, which is the providing of continuous monitoring. Conventional clinical practices often allow for discrete, scheduled medical consultations, leaving gaps in patient care when immediate attention is most needed. The IMIDoc app was designed to bridge this gap, ensuring timely medical oversight of patients.

#### Primary Source of Financing

This project was financially supported by a grant from the Fondo de Investigaciones Sanitarias (FIS) of the Instituto de Salud Carlos III (PI22/00777). The grant specifically facilitated the technical development of our digital solution and will allow us to carry out the clinical-technical evaluation in a clinical study.

#### Analysis of Previous Experience and Unmet Needs

In the platform ideation phase, relevant aspects and features that the digital solution should incorporate were identified. During this phase we considered the opinion of 41 patients and 4 clinicians about their experience with the previous digital solution (Table S1 in Multimedia Appendix 1). Regarding the patients, usability ranged from 85.3%‐97.5%; most found the digital solution easy to use, and well adapted. Over 90% (38/41) of participants found the application was well adapted to its objective, with an adequate level of detail. Most patients reported considered that it allowed them to record their current health situation and would recommend it. The areas patients most frequently cited as needing improvement were the application’s design and the amount and detail of informational content related to the disease. On the other hand, clinicians rated the overall usability and design (0‐10, from worse to best) of the application with a mean (SD) of 8.50 (1.22), the app utility with 8.50 (1.16), and the feasibility with 7.56 (2.41). They primarily expressed concerns regarding the feasibility of the implementation in clinical practice, particularly in terms of time and logistics, followed by the performance of the solution.

### Development Phase

The iterative development process was carried out over 15 sprints from January to August 2023, each punctuated by a bi-weekly update meeting and culminating in a final review meeting (Supplementary Figure 1 in Multimedia Appendix 1). The initial meetings laid the groundwork by focusing on service architecture and basic functionalities like login and user registration. Subsequent meetings were geared toward enhancing the user interface, developing workflows in the digital solution, and integrating questionnaire designs. In these bi-weekly review meetings, the development team assessed progress and recalibrated, when necessary, thereby allowing them to respond to changing requirements and deliver a high-quality product efficiently. During each sprint, different tasks were assumed by the development team with the aim of achieving the sprint goal, which was previously set in an online meeting by the clinical team. The middle phase emphasized refining communication features, resource and medication development, and incident management. Data visualization tools like historical graphs and evolution screens were also integrated. The concluding meetings targeted system debugging, specifically in messaging and error management, before a comprehensive final review was conducted.

The collaborative and interdisciplinary process made it possible not only to define the instruments to be implemented for the collection of data of interest, but also to design and develop the applications for the users; that was , mobile apps for patients with RA or SpA, and a Web application for clinicians.

#### Set of Variables and Instruments

In accordance with the objectives of the clinical study, in which the implementation of the MAM model in clinical practice through IMIDoc will be evaluated, Textbox 1 specifies the clinical and ePROS variables that the solution was designed to capture and that comprise the monitoring and follow-up of patients with RA and SpA.

In addition to the capture of clinical variables, instruments have been implemented to evaluate the usability of the applications, satisfaction, and user experience: System Usability Scale (SUS) and the Net-Promoter Score (NPS) indicator [18,19]. These were evaluated at 10 weeks and 34 weeks, respectively.

Textbox 1. **Clinical variables and electronic patient-reported outcomes collected by the IMIDoc app.**

1) General characteristics

- Sociodemographics and treatment information including age, sex, toxic habits, date of diagnosis, date of initiation of treatment, medication, etc.
- Electronic patient-reported outcomes (ePROs)
  a) Patient Global Health (PGH)
  b) Visual Analogue Scale (VAS)
  c) Tender Joint Count (TJC) and Swollen Joint Count (SJC)
- Events or incidents
  a) Flare
  b) Infection
  c) Medication problem
  d) Other reasons
- Adherence

  a) Compliance Questionnaire on Rheumatology, CQR

2) Rheumatoid arthritis (ePROs)

- Disease Activity Score 28 (DAS28)
- Health Assessment Questionnaire (HAQ)

3) Spondyloarthritis (ePROs)

- Bath Ankylosing Spondylitis Disease Activity Index (BASDAI)
- Axial Spondyloarthritis Disease Activity Score (ASDAS)
- Assessment of SpondyloArthritis International Society Health Index
- Painful enthesis count

#### System Architecture

**Figure 1.**
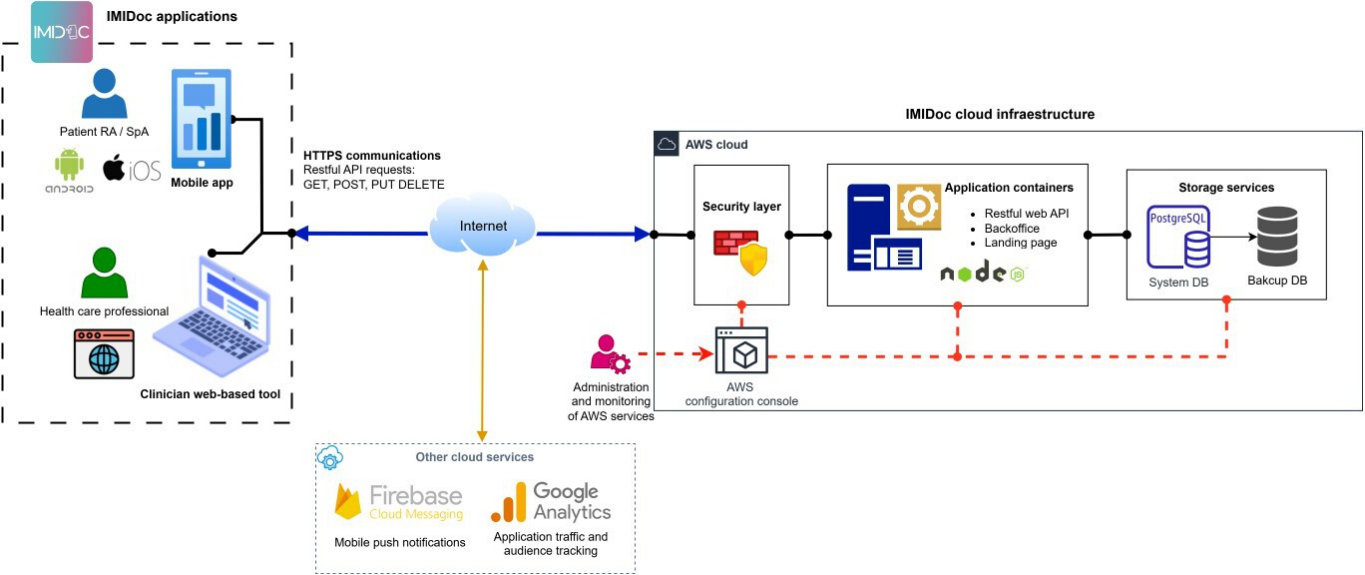
Diagram of the architecture of the IMIDoc solution. RA: rheumatoid arthritis; SpA: Spondyloarthritis; AWS: amazon web services; DB: database; API: Application Programming Interface.

The analysis and design phase of the project made it possible to identify the user profiles and the functionalities and characteristics of the applications included in the IMIDoc digital health solution. Figure 1 shows a schematic of the main agents, components, and services of the system architecture using the MAM applied to RA and SpA. The figure shows the users (patients and health care professionals) accessing their respective applications and communicating with the service infrastructure deployed in the cloud.

#### IMIDoc Applications

The first steps in the development of the IMIDoc applications (the mobile app for patients and the web interface for professionals) were focused on addressing the interface design. For this purpose, we used the Figma platform, which is a cloud prototyping platform that facilitates collaborative work.

**Figure 2.**
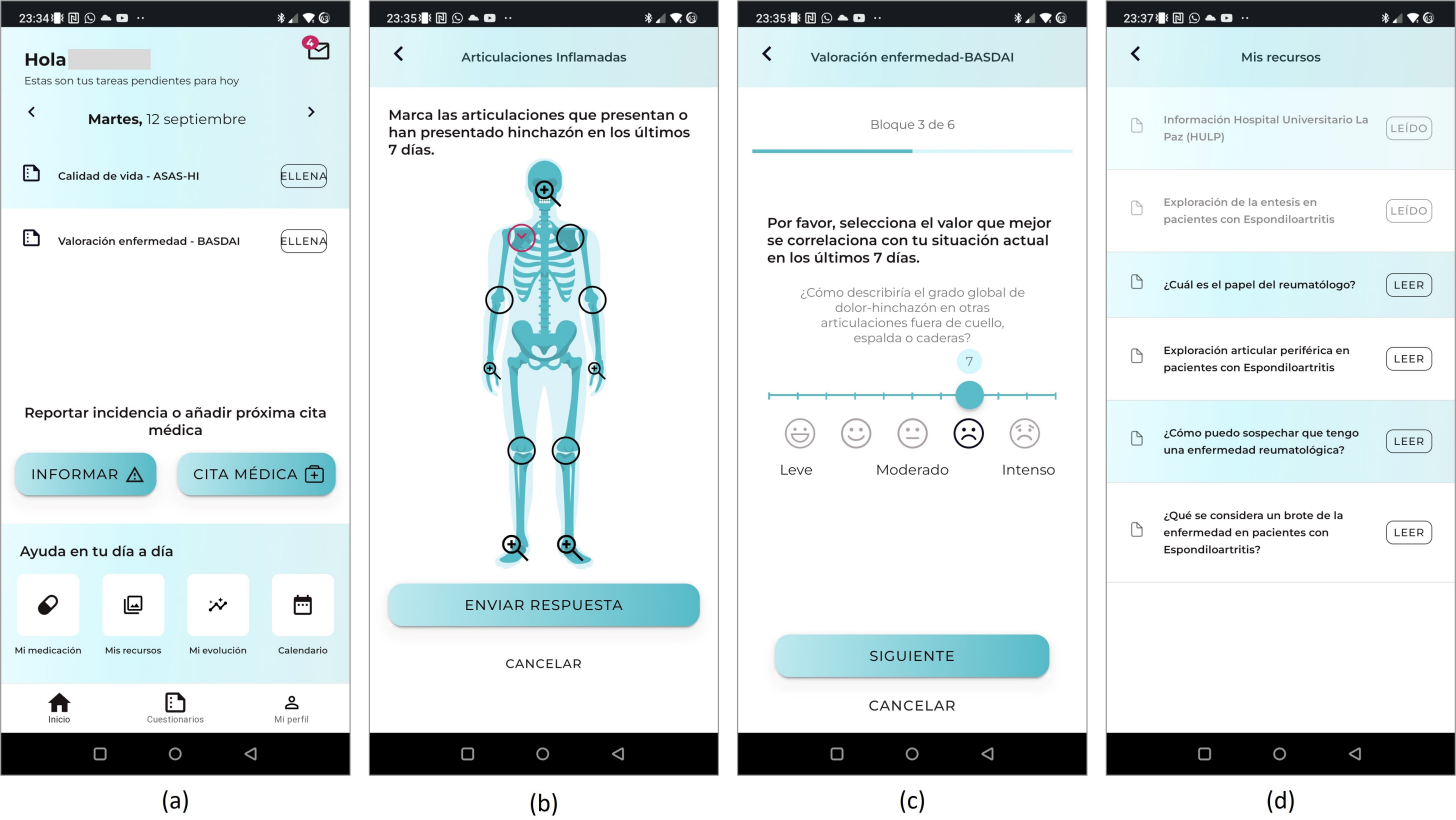
Screenshots of the Spanish version of IMIDoc app designed for patients with rheumatoid arthritis (RA) and spondyloarthritis. (a) Home screen displaying pending tasks, including daily questionnaires, and options to report incidents, access medication details, educational resources, disease progression, and a calendar. (b) Interface for marking swollen joints by patient's perception. (c) Example of a Bath Ankylosing Spondylitis Disease Activity Index (BASDAI) score questionnaire item for disease assessment. (d) List of informational and educational resources available within the app.

The IMIDoc mobile app (Figure 2) was designed to incorporate functionalities for recording disease activity, as well as some other domains potentially affected by RA or SpA.

In addition, the IMIDoc app included scenarios for medication management. To achieve this, the professional team carefully consulted the latest clinical practice guidelines for RA and SpA. This comprehensive review of current guidelines informed the creation of tools for monitoring disease activity and managing medication within the app.

A section with educational and informative content to help patients understand their disease and treatment options was also included; specifically, 26 educational articles were generated by clinicians with multimedia components, images and 12 educational videos. Specialists in various fields, including immunology and obstetrics, were engaged to contribute to the development of educational resources. The material produced included illustrated resources designed to improve patient understanding of their condition and the treatment options available. In addition, a diverse range of formats was used to adapt to different preferences. This included the creation of infographics, interactive texts, and videos. Each format was carefully selected to best suit the content being presented, with the aim of enhancing user engagement and understanding.

Another feature of the app involves task and medical appointment reminders in a personalized questionnaire that is complemented by push notifications.

**Figure 3.**
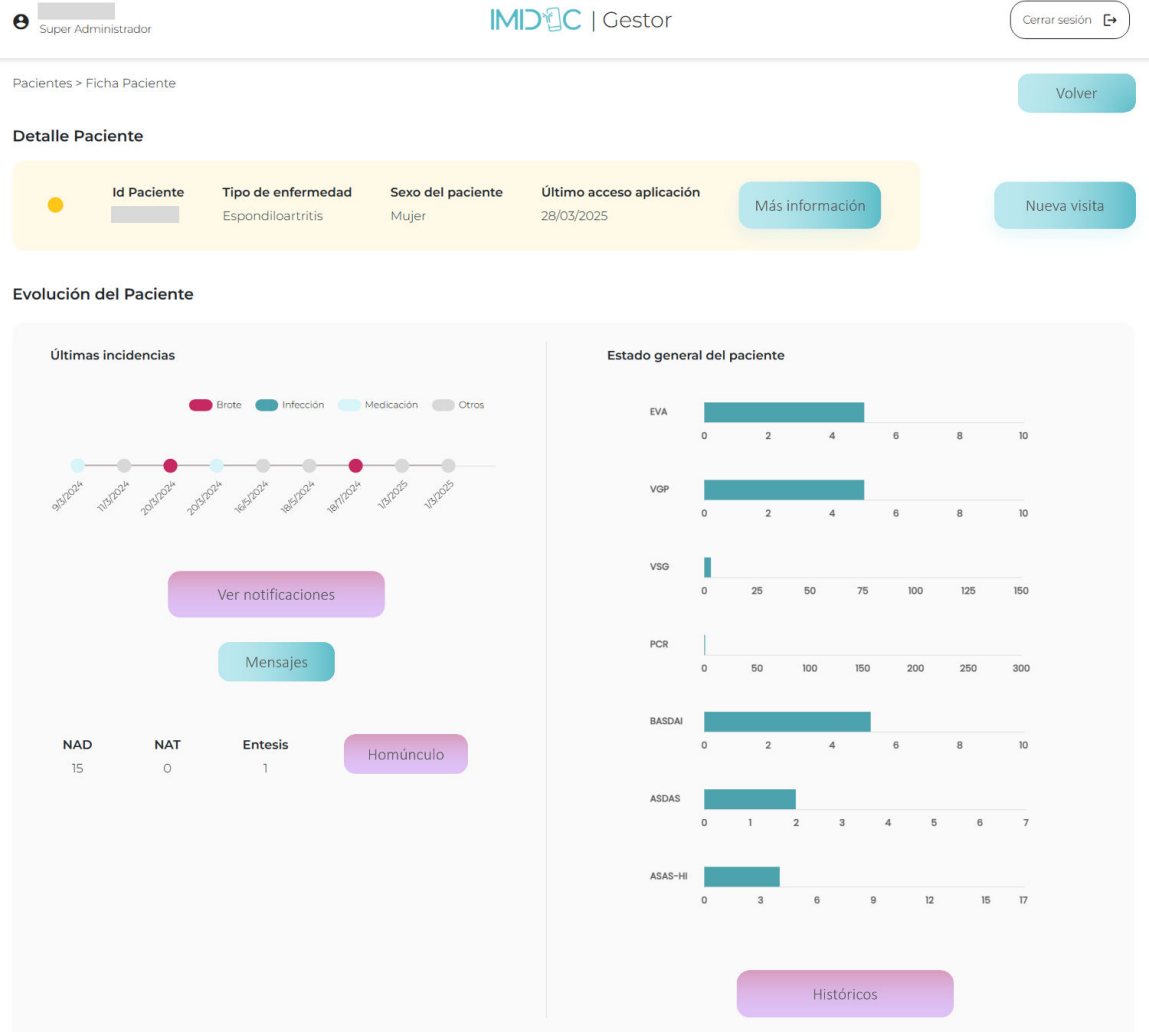
Screenshot of the Spanish version of IMIDoc web-based tool for monitoring patients with rheumatoid arthritis and spondyloarthritis.

The IMIDoc clinician web platform is a web-based application for health care professionals, primarily clinical rheumatologists [21]. The IMIDoc clinician web platform includes scenarios for monitoring ePROs in a prespecified time frame and generates events or alerts. These features will enable clinicians to respond in near real-time to significant changes in disease activity. Figure 3 shows an image of the screen displaying the data recorded by a patient.

The top section displays basic patient information, including patient code, sex, and last application access. Below, the left panel shows recent incidents (flares, infections, medication issues, or others), the number of swollen joints (NAT, Spanish acronym for “Número de Articulaciones Tumefactas”), the number of tender joints (NAD, Spanish acronym for “Número de Articulaciones Dolorosas”), enthesitis, and access to a homunculus visualization, along with options to view patient messages. The right panel below features bar graphs tracking patient progress, including pain scale (EVA, Spanish acronym for “Escala Visual Analógica”), patient global assessment (VGP, Spanish acronym for “Valoración Global del Paciente”), erythrocyte sedimentation rate (VSG, Spanish acronym for “Velocidad de Sedimentación Globular”), C-reactive protein level (PCR, Spanish acronym for “Proteína C Reactiva”), Bath Ankylosing Spondylitis Disease Activity Index score (BASDAI), and Axial Spondyloarthritis Disease Activity Score (ASDAS).

**Figure 4.**
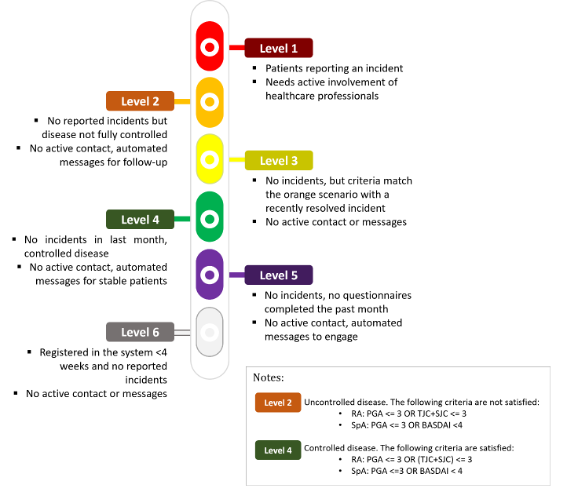
Classification of the health status and clinical adherence of patients with rheumatoid arthritis and spondyloarthritis. PGA: patient global assessment, TJC: tender joint count, SJC: swollen joint count, BASDAI: bath ankylosing spondylitis disease activity index.

Through IMIDoc, clinicians could quickly identify the health status of patients and their level of adherence based on the automatic processing of data. The IMIDoc classification algorithm assigns 6 different levels to patients as shown in Figure 4.

#### IMIDoc Backend

As mentioned above, there are several platforms available from technology providers such as Amazon Web Services (Amazon), Azure (Microsoft), Google Cloud (Google) and IBM Cloud Computing (IBM), among others. For the implementation of IMIDoc, we selected Amazon Web Services (AWS) to deploy the backend components. In addition, AWS offered a number of features of interest for our implementation [15,16]. We used AWS Fargate which is a serverless computing engine for containers, in which a Node.js-based application has been deployed that features a RESTful API to respond to requests using GET, POST, PUT, DELETE methods via HTTPS. For the storage of ePROs data and clinical actions, a relational database PostgreSQL with Amazon RDS was implemented and deployed in the European region (Spain). An important aspect to consider was security, for which the Security Hub service was enabled. This integrates functionalities to detect and manage security events, in addition to other components such as a Firewall and distributed denial of serviceprotection. Moreover, AWS complies with various certifications, accreditations, European laws and regulations such as ENS (high category), DESC CSP, CISPE and the General Data Protection Regulation (GDPR). It should be noted that these characteristics, in terms of security and data processing, are requirements mandated by the Information Systems Security Office for the approved use of the application in health care. Finally, AWS allows us to use a continuous integration and delivery service and, in this way, allows us to maintain and prepare system updates for subsequent release in production.

The system has modules that run automatically, monitoring the data, establishing the patient’s health status, and generating notifications that are sent to the patients’ app through the Google Firebase service. Table 1 specifies the types of notifications included in IMIDoc.

Google Analytics was used to track and process dimensions and metrics related to the use of both applications: country, device, operating system, app version and user interactions with the app (number of sessions, duration of sessions, screen views, and interaction times). Google Analytics service and mechanisms were integrated to record the usage metrics of both applications.

**Table 1.** IMIDoc mobile app notifications.

Dimension	Trigger	Title	Body
Messages	Response from a health care professional to an incident.	IMIDoc^a^ notification	*“*You have a new message*”.*
Medication	Time of medication intake	IMIDoc medication	*“*You have medication pending confirmation*”*.
Resources	New reading resource available	IMIDoc multimedia content	*“*You have new educational resources available*”*.
Questionnaire(s)	New questionnaire(s) to fill out	IMIDoc questionnaires	*“*You have new questionnaire(s) to fill in*”*.
Medical appointment	One day before the medical appointment	IMIDoc medical appointment	*“*Remember that you have a doctor’s appointment tomorrow, please check that you have provided all the necessary information”*.*
Adherence support	One week without logging into the app	IMIDoc misses you	*"*We have noticed that you have not been using the application for a while, we encourage you to give us another chance*”*.

a IMIDoc: Immune-mediated inflammatory diseases

### Preclinical Testing Phase of IMIDoc

The involvement of the multidisciplinary team in the initial testing phase led to several modifications to the digital solution, primarily to the mobile solution. These mainly focused on the user interface, the wording used within the application, and its distribution mechanisms, aiming to ensure that the application was intuitive for both patients and health care professionals.

During the subsequent 12-week digital solution testing phase with 10 active users (7 professionals and 3 patients), several functional issues and areas for potential improvement were identified. Detailed functionality reports for the mobile app recorded 111 entries—76 errors, 16 improvements, and 10 evolutionary suggestions—while the professional tool generated 45 entries, with 40 errors and 5 evolutionary suggestions. Based on this feedback, users’ reported difficulties with navigation and data entry prompted a redesign of the interface to reduce logging steps and incorporate clearer prompts. In addition, the need for customizable alerts led to refinements in medication and appointment reminders and improvements in the messaging system to enable prompt clinician responses. These iterative enhancements, delivered over 10 update cycles, set the stage for a multicenter clinical study involving 360 patients from 5 Spanish hospitals.

## Discussion

This study underscores the efficacy of using a structured cocreative approach to crafting the IMIDoc digital health application tailored for rheumatology practice. Over the years, the rheumatology community has voiced the demand for refined apps and tools to yield meaningful clinical outcomes [20,22]. Despite advancements in mobile health technology (mHealth) and treatment in rheumatology, there persists a notable gap in continuous patient monitoring [23,24]. Recognizing this unmet need in rheumatology care, particularly during intervals between scheduled consultations, informed the conceptualization of the digital health solution presented here.

Our initial needs assessment drew from past experiences, incorporating feedback from clinicians and patients to identify key features and functionalities of IMIDoc [6]. This information informed requirements and specifications, supporting a participatory design approach complemented by UCD principles to enhance usability, acceptability, and user experience [17]. Embracing such techniques is crucial for crafting more effective health interventions as it incorporates diverse perspectives [25]. A recent systematic review underscores the increasing adoption of cocreated technology solutions in health care [26]. This aligned with our own approach in developing IMIDoc, in which the representation of both patients and clinicians ensured that real needs were met. The involvement of all relevant stakeholders, including biomedical engineers and technicians, throughout design and development fostered communication, transparency, and a design that aligned with users’ priorities.

Furthermore, the application of agile methodologies during the development phase, with iterative cycles, proved invaluable. This approach facilitated continuous adjustments and input, minimizing risks and adapting to emerging changes, and proved particularly valuable in a rheumatology clinical setting [27]. While previous literature has acknowledged the benefits of Agile methodologies [28], our study empirically substantiates its efficacy in this specific context. The pilot testing phase provided valuable insights and led to necessary adjustments, highlighting the dynamic and responsive nature of a development cycle based on agile methodologies. As an example, participants frequently reported difficulties in navigating the app and entering data. In response, the interface was redesigned to minimize the number of steps needed to log information and incorporate clearer prompts to guide users. Additional feedback highlighted the need for customizable alerts, leading us to refine medication and appointment reminders, and to improve the messaging system so clinicians could rapidly respond to patient messages.

Challenges in managing the project scope and addressing various complexities led to a shift toward a more strategic and modular approach, prioritizing essential features and refining functionalities based on end-user needs. To ensure a rigorous assessment of usability and patient satisfaction, instruments such as the SUS and NPS have been implemented and will be applied in the upcoming multicenter clinical study involving 360 patients from 5 Spanish hospitals.

To ensure the long-term sustainability and continuous improvement of IMIDoc, the project team is actively pursuing additional funding avenues beyond the initial grant. This includes applying for public research grants, seeking private partnerships with health care technology companies, and exploring collaborations with pharmaceutical firms interested in supporting digital health initiatives. In addition, we are developing a strategic plan that outlines regular updates and maintenance schedules for the IMIDoc platform to keep pace with technological advancements and evolving clinical needs. By diversifying our funding sources and establishing a robust maintenance framework, we aim to maintain the app’s functionality, incorporate user feedback, and expand its features to better serve patients and health care providers in the future.

Previous literature has provided insights into patient perspectives on digital health interventions and has revealed diverse opinions among patients. Moreover, it has highlighted both advantages and concerns regarding telemedicine in rheumatology. A mixed-methods approach study that explored the acceptability and safety of telemedicine in rheumatology during the COVID-19 pandemic highlighted the need for careful patient selection and further consultation with clinicians to improve telemedicine’s acceptability and safety [29]. A prospective study including patients referred to a Veterans Affairs rheumatology clinic found that telerheumatology was well-received by patients, and accurately identified all inflammatory rheumatic conditions at the telerheumatology visits. This suggests that remote monitoring may be suitable for screening and prioritizing patients for in-person rheumatology clinics [30]. Recent research has demonstrated the potential for digital tools to yield positive outcomes in health care. Two randomized trials have been conducted in RA. Pers et al [31] compared a smartphone application connected to a physician’s interface for monitoring RA with conventional monitoring. The connected monitoring group had significantly fewer physical visits while maintaining tight control of disease activity and improving quality of life [31]. Salaffi et al [32] investigated the effectiveness of a telemonitoring strategy in early RA, showing higher rates of remission by CDAI within a 1 year compared with the conventional group (38.1% vs 25%) [32].

Our approach has certain limitations. At present, the focus is restricted to RA and SpA, which may limit the generalizability of IMIDoc to other rheumatologic disorders. The small number of participants in the pilot, without extensive demographic characterization, constrains broad interpretations of feasibility and usability. Continued funding is crucial for further feature enhancements and to maintain robust data protection measures, ensuring compliance with evolving regulations. Future large-scale evaluations are planned to validate the efficacy of IMIDoc, broaden its applicability to wider patient populations, and strengthen its long-term sustainability.

While patient opinions on telemedicine may vary, these studies have shown the potential for digital tools to improve clinical outcomes, such as achieving disease remission and reducing clinical visits. Therefore, it is essential to embrace these technological advancements by implementing digital health solutions in real-world rheumatology practice. To this end, it is essential to carefully select patients, and work closely with clinicians to ensure that digital health interventions are effectively integrated into clinical practice, ultimately enhancing the care and well-being of rheumatology patients.

### Conclusions

The development of IMIDoc underscores the significance of a cocreative design approach in health care, on that can lead to a product that not only excels in functionally, but that is also contextually relevant to rheumatology practice. Addressing unmet needs such as the integration of ePRO collection, tailored alerts, and a robust messaging system into a single platform, IMIDoc provides a digital health platform for comprehensive disease activity tracking, medication management, patient education, and empowerment in self-health management. This innovation is pivotal to navigating the complexities of managing patients with immune‐mediated inflammatory diseases, offering personalized and effective management strategies while reducing the gap between clinic visits. This work strives to be a breakthrough in rheumatology care, and the proposed multicenter study aims to further validate and support these findings.

## Supplementary material

10.2196/58095 Multimedia Appendix 1 Patient questionnaire.

## Abbreviations

AWS: Amazon Web Services
ePROs: electronic Patient-Reported Outcomes
GDPR: General Data Protection Regulation
MAM: Mixed Care Model
mHealth: mobile health
NPS: Net-Promoter Score
RA: rheumatoid arthritis
SUS: System Usability Scale
