# Supplementary material for: Digital Health Intervention for Patient Monitoring in Immune-Mediated Inflammatory Diseases: Cocreation and Feasibility Study of the IMIDoc Platform
Source: JMIR Hum Factors. 2025 Apr 21;12:e58095. doi: 10.2196/58095 (PMC12036949; doi:10.2196/58095)
Supplement: Multimedia Appendix 1 [file humanfactors-v12-e58095-s001.docx]

## **Supplementary text 1:**

**Patient Questionnaire**

The patient interview was designed to assess the usability and utility of the application from a patient's perspective. The interview was divided into two main sections:

Usability:

- Ease of Use: "Is the application easy to use?"
- Adaptation of Functions: "Are the different functions of the digital solution well adapted?"
- Design Aesthetics: "Does the app have a nice design?"
- Design Appropriateness: "Does the design fit the purpose of the application?"

Usefulness:

- Usefulness of Content: "I found the content of the application useful."
- Adequacy of Detail: "The application's level of detail seemed adequate to me."
- Recording Health Situation: "I believe that the content of the application allows me to record my current health situation."
- Recommendation to Other Patients: "I would recommend this app to a patient with RA/SpA."

The following questions were posed to elicit patients’ open feedback: i) general usability and design of the application: "Do you have any general comments on how easy it was to use the application or on its design?"; ii) Preferred feature: "What did you like most about this application?"; iii) least liked feature: "What did you like least about this application?"; iv) suggestions for improvement: "Do you have any suggestions for this application?"

**Clinicians' Questionnaire**

The clinicians' questionnaire was designed to evaluate the usability, utility, and feasibility of the application in clinical practice. The questionnaire consisted of several sections, each addressing different aspects of the application:

Usability and Design of the Application:

- Ease of use of the clinician interface: "How easy did you find the clinician interface to use?"
- Aesthetic appeal of the clinician interface: "How would you rate the design appeal of the clinician interface?"
- Adaptation of questionnaires and content for ease of completion and understanding: "Do you think the questionnaires and included content is well adapted for easy completion and understanding?"
- User-friendliness of the design: "How user-friendly did you find the design of the application?"
- Technical functionality: "How would you rate the technical functionality of the solution?"

Usefulness of the Application:

- Usefulness of the application's content in clinical decision-making: "How useful did you find the application's content for your clinical decisions?"
- Helpfulness of questionnaire frequency: "How helpful did you find the frequency of the questionnaires?"
- Impact on consultation efficiency: "Do you think the application improves your efficiency during consultations?"
- Contribution to understanding patient disease progression: "Do you believe the information obtained from the application enhances knowledge about the real progression of patients’ diseases?"
- Recommendation to peers: "Would you recommend this application to your colleagues?"

Feasibility in Clinical Practice (Scored 0-10):

- Time efficiency in reviewing the medical interface: "Do you find that reviewing the information provided by medical interface could be done in a time-efficient manner?"
- Time needed to resolve recorded medical incidents: "How quickly were you able to resolve recorded medical incidents?"
- Feasibility of implementing such a tool within the organization's current administrative infrastructure: "Do you consider it feasible to implement a tool of this nature within the current logistic organizational infrastructure of your center?"
- Need for a specific consultation for implementation: "Do you believe that the implementation of this care model requires a consultation to specifically review the results?"

Open Comment A: "Do you have any general comments on the use of the clinician interface or its design? And on the patient interface?"

Comment B: "Rank from 1 to 7 (1 being the most relevant) the main barriers professionals face when using telehealth care models in clinical practice:"

- Lack of interest in this topic.
- Shortage of personnel dedicated to this field.
- Lack of training in this field.
- Time constraints within the current administrative structure of your organization.
- Distrust of digital tools.
- Scarcity of useful digital tools in clinical practice.
- Other causes (please specify).

Open Comment C: "What did you like most about the clinician interface?"

Open Comment D: "What did you like least?"

Open Comment E: "Do you have any final comments on this application?"

## **Supplementary text 2:**

### Technical details

In this section, the technical details pertain to the underlying technological framework of the IMIDoc system, focusing on its client-server architecture, the utilization of cloud computing to enhance healthcare delivery, and the development methodologies for patient and healthcare professional interfaces across various platforms and devices.

The model selected for the architecture of IMIDoc was client-server based. This architecture allows for distributed computing between two entities: the client and the server (1). In the case of IMIDoc, the "client" corresponds to both patients and healthcare professionals using physical smart devices and computers software interfaces, respectively. On the other hand, the server(s) consists of the infrastructure that not only processes and stores information, but also responds to clients’ requests. Among the advantages of this type of architecture are its scalability, ease of administration, security, and the possibility of providing health services through mobile devices. This approach helps to provide more accessible and efficient healthcare (2, 3).

There has been an increasing interest and use of cloud-based computing services in the healthcare sector for some years now, with a particular focus on citizen-centric services and system interoperability (4, 5). Essentially, this is because the cloud computing paradigm offers alternatives facilitating the optimization, efficiency and scalability of resources, as well as their customization and adaptability, all of which helps to minimize costs (6, 7). In the literature, several authors have concluded that the need for computational and communication resources has emerged as the main reason for using the cloud in healthcare. This explains why there are a significant number of applications that combine mHealth with cloud-based computing and/or services (8, 9, 10).

The term 'cloud computing' started to become popular with the commercialization of such services by large companies such as Amazon, Google and Microsoft. It refers broadly to a network of servers or remote services hosted in different locations and accessible via the Internet, although it can also signify a private network used to store, manage and process data in place of locally managed resources. These remote servers or resources can offer a host of different capabilities, whereby the provider can make available to customers an infrastructure consisting of a physical or virtualized server (Infrastructure as a Service, IaaS). It can also allow customers to abstract even from the code and can offer specific functionalities in the form of services (Platform as a Service, PaaS or Software as a Service, SaaS).

In this context, patients (clients) in their daily environment will use the services offered via their mobile devices. The development of the patient applications has become increasingly common on Android and iOS platforms. Currently, both platforms command the entire market share. Android is the most widely used operating system worldwide, with a market share in Q3 2023 of 70.5%. In contrast, iOS accounts for 28.8% of the global market (11). Each platform has its own specific integrated development environment (IDE) and programming languages. To develop Android applications, Java and Kotlin languages are used with the Android Studio IDE. For their part, iOS applications are mainly programmed in Objective-C and Swift languages through the Xcode IDE.

The use of different programming languages and environments may result in higher production costs due to the need for teams of developers specialized in each platform. In addition, the costs associated with the maintainability and scalability of the application can also increase.

A similar situation can be found in the world of healthcare professionals (clients), who normally use desktop computers in medical consultation rooms. There are different operating systems and platforms that can involve specific developments. However, in the case of desktop or laptop computers, nowadays it is common to use Web applications that facilitate accessibility from any type of device.

## **Supplementary Table 1.**

| Patient Interview | | | Clinician Interview | |
| --- | --- | --- | --- | --- |
| Usability | | | Usability and design | 0 to 10 |
| Is the application easy to use? | | 97.5% | I find the doctor interface easy to use | 8.50 ±- 1.29 |
| Are the different functions of the digital solution well adapted? | | 97.5% | The doctor interface has a nice design | 8.75 ±- 0.95 |
| Does the app have a nice design? | | 85.3% | I think that the questionnaires and content included are easy to understand and complete | 8.75 ±- 0.95 |
| Does the design fit the purpose of the application? | | 95.1% | The design is user-friendly | 8.50 ±- 1.29 |
| Usefulness | | | The technical performance of the solution is adequate | 8.00 ±- 1.63 |
| I found the application's content useful. | | 97.5% | Utility | 0 to 10 |
| The application's level of detail seemed adequate to me. | | 92.7% | I found the content of the application useful for making clinical decisions | 8.50 ±- 1.29 |
| I think that the content of the application allows me to accurately record my current health situation. | | 90.2% | The frequency of the questionnaires seemed adequate to me | 8.75 ±- 0.95 |
| I would recommend this app to a patient with RA/SpA. | | 97.5% | I consider that the application improves my efficiency during consultations | 8.75 ±- 0.95 |
|  |  |  | I think that the information obtained from the application helps me better assess the patient's real disease evolution | 8.50 ±- 1.00 |
|  |  |  | I would recommend this app to other colleagues | 8.00 ±- 1.63 |
|  |  |  | Feasibility in clinical practice | 0 to 10 |
|  |  |  | I find reviewing the information provided by medical interface to be tine-consuming | 8.50 ±- 0.57 |
|  |  |  | i was able to rapidly resolve the registered medical incidents | 7.50 ±- 2.51 |
|  |  |  | I think implementing such at my current center is feasible | 7.50 ±- 2.51 |
|  |  |  | I believe that implementation of this care model requires a consultation to specifically review the results | 6.75 ±- 4.03 |


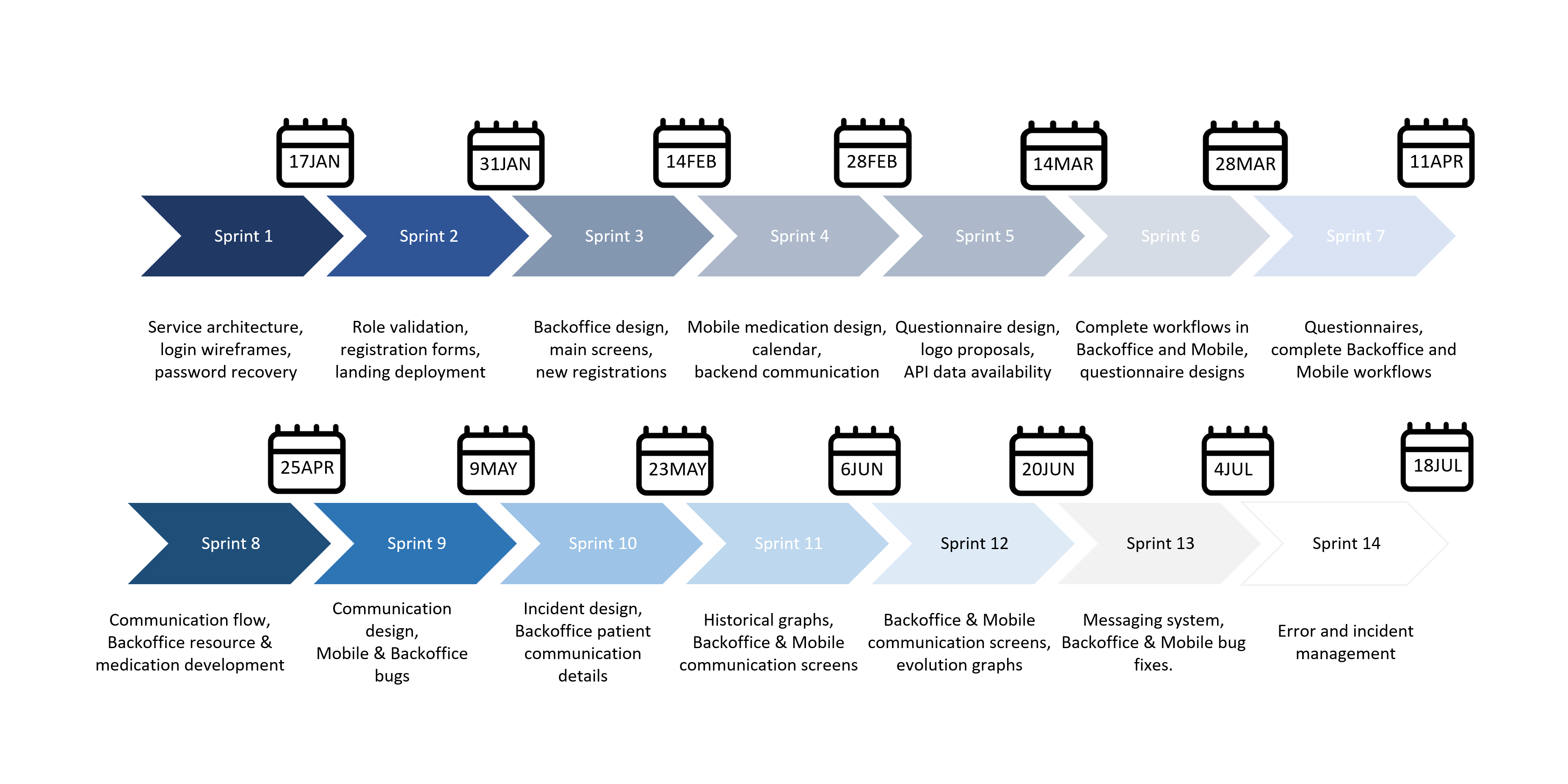


**Supplementary Figure 1. Development stages of the digital health solution in the year 2023**

**References**

1. Kumar, S. (2019). A review on client-server based applications and research opportunity. International Journal of Recent Scientific Research, 10(7), 33857-3386.
2. Rashid Bashshur, Gary Shannon, Elizabeth Krupinski, and Jim Grigsby. The Taxonomy of Telemedicine. Telemedicine and e-Health.Jul 2011.484-494.http://doi.org/10.1089/tmj.2011.0103
3. Richard K. Lomotey and Ralph Deters. 2013. Efficient mobile services consumption in mHealth. In Proceedings of the 2013 IEEE/ACM International Conference on Advances in Social Networks Analysis and Mining (ASONAM '13). Association for Computing Machinery, New York, NY, USA, 982–989. <https://doi.org/10.1145/2492517.2500279>
4. Omar Ali, Anup Shrestha, Jeffrey Soar, Samuel Fosso Wamba, "Cloud computing-enabled healthcare opportunities, issues, and applications: A systematic review", International Journal of Information Management, Volume 43, 2018, Pages 146-158, ISSN 0268-4012, <https://doi.org/10.1016/j.ijinfomgt.2018.07.009>.
5. Casola V, Castiglione A, Choo KKR, Esposito C. Healthcare-Related Data in the Cloud: Challenges and Opportunities. IEEE Cloud Computing. 2016 Nov 1;3(6):10–4.
6. Chung, K., Park, R.C. ARTÍCULO RETRACTADO: Red u-healthcare basada en la nube con garantía de QoS para el servicio de salud móvil. Cluster Comput 22 (Supl 1), 2001–2015 (2019). <https://doi.org/10.1007/s10586-017-112>
7. Enrique Gonzalez, Raul Peña, Alfonso Avila, Cesar Vargas-Rosales, David Munoz-Rodriguez, "A Systematic Review on Recent Advances in mHealth Systems: Deployment Architecture for Emergency Response", Journal of Healthcare Engineering, vol. 2017, Article ID 9186270, 13 pages, 2017. https://doi.org/10.1155/2017/9186270
8. Boyi Xu, Lida Xu, Hongming Cai, Lihong Jiang, Yang Luo & Yizhi Gu (2017) The design of an m-Health monitoring system based on a cloud computing platform, Enterprise Information Systems, 11:1, 17-36, DOI: [10.1080/17517575.2015.1053416](https://doi.org/10.1080/17517575.2015.1053416)
9. Mehmood I, Sajjad M, Baik SW. Mobile-Cloud Assisted Video Summarization Framework for Efficient Management of Remote Sensing Data Generated by Wireless Capsule Sensors. *Sensors*. 2014; 14(9):17112-17145. <https://doi.org/10.3390/s140917112>
10. Laura García, Jesús Tomás, Lorena Parra, Jaime Lloret, "An m-health application for cerebral stroke detection and monitoring using cloud services", International Journal of Information Management, Volume 45, 2019, Pages 319-327, ISSN 0268-4012, https://doi.org/10.1016/j.ijinfomgt.2018.06.004.
11. Global market share held by mobile operating systems from 2009 to 2023, by quarter. Published by Statista Research Department, Oct 4, 2023. <https://www.statista.com/statistics/272698/global-market-share-held-by-mobile-operating-systems-since-2009/>
